# The Relation between Different Aspects of Quality of Life with Coping Style in Adolescents with Thalassemia in Comparison to a Healthy Group

**Published:** 2020-01-01

**Authors:** Samira Abbasi, Mohsen Shahriari, Majid Ghanavat, Sedigheh Talakoub, Fatemeh Sadat Mosavi Asl, Zeinab Hemati

**Affiliations:** 1 Department of Psychiatric Nursing, School of Nursing and Midwifery, Isfahan University of Medical Sciences, Isfahan, Iran; 2 Department of Adult Health Nursing, School of Nursing and Midwifery, Nursing and Midwifery Care Research Centre, Isfahan, Iran; 3 Child Growth and Development Research Center, Isfahan University of Medical Sciences, Isfahan, Iran; 4 Department of Pediatrics, School of Nursing and Midwifery, Isfahan University of Medical Sciences, Isfahan, Iran; 5 NICU Ward, Gharazi Hospital, Isfahan, Iran; 6 Department of Pediatrics, School of Nursing and Midwifery, Nursing and Midwifery Care Research Center, Isfahan University of Medical Sciences, Isfahan, Iran

**Keywords:** Thalassemia, Adolescents, Quality of life, Coping style

## Abstract

**Background:** Thalassemia as a chronic disease could affect different aspects of a patient’s life. On the other hand, when encountering the symptoms of a chronic disease as a stressful factor, the coping strategy of the adolescents and their families could have an important role in the quality of life of these patients. The present study was conducted to determine the relation between different aspects of quality of life with coping styles in the adolescents with thalassemia in comparison to a healthy control group.

**Materials and Methods**: The present study is a case-control research in 2017. Studied samples were 200 adolescents with thalassemia and healthy adolescents. Data gathering tools were demographic characteristics checklist and the coping style and quality of life questionnaire by the World Health Organization. Data were analyzed by SPSS 20 using independent *t*-test, linear regression and correlation coefficients.

**Conclusion:** Results of Pearson statistical test showed a positive and significant relation between the total mean score of quality of life and its physical, social and mental aspects with emotion-oriented coping style (p<0.01). Also a direct significant relation was observed between the total mean score of quality of life and its social and physical aspects with problem-oriented coping style(p<0.01).

**Conclusion: **According to the results of the present study, educating the adolescents and their families for paying attention to the coping style for stressful factors and preparing these adolescents for passing toward the youth period, which could be challenging for them, are highly recommended.

## Introduction

 Thalassemia is a genetic blood disorder which is characterized by a decrease in the globin chain synthesis or non-synthesis. This synthesis defect leads to the formation of abnormal and brittle red blood cells which can be easily hemolysed and lead to chronic anemia. The World Health 

Organization (WHO) identifies thalassemia as the most common genetic blood disorder, in more than 60 countries with a population of more than 150 million people. This disorder is usually prevalent among children in the Middle East, the Mediterranean, and South part of Asia ^1^.

There are more than 20,000 patients with B-Thalassemia in Iran, with an incidence of 4-8 per 1000 births. When they get older, these patients are faced with new socio-cultural problems such as lack of proper occupation, marital problems, ongoing hospital visits, poor mental images caused by their inappropriate appearance, bone deformity and limited social activities, which cause plenty of mental-psychiatric disorders for them, and affect their quality of life^1^^,^^2^. According to the definition presented by WHO, quality of life means people's perception of their position in life based on the value and cultural systems in which they live and is related to their goals, expectations, stresses and concerns^3^. Sultana et al. showed that thalassemia is inversely affecting the physical and mental health of children. The results of studies on the quality of life in patients with chronic diseases show that to treat these diseases, the adaptation to the disease and the self-care play a key role in therapeutic outcomes; and in this field, just having information is not enough, but the patient's ability to effectively deal with the stresses of chronic diseases in everyday life is the most important point ^4^^, ^^5^.

Coping strategies are rational and conscious methods for regulating negative emotions. Based on psychological theories, these strategies play an important role in managing stressful situations, reducing stress, and ultimately in mental health and psychological and physical well-being of individuals^5^. Although coping strategies, either active or inactive, include a set of efforts which individuals make to avoid health-threatening factors, using any one of these styles does not necessarily lead to the reduction of psychological factors; the effectiveness of coping styles is different in different situations and sometimes they not only do not reduce the stress, but also increase it ^6^. 

Jovic et al. (2016) showed that adolescents with diabetes type 1 reject most of coping passive ways such as social isolation, by using active coping methods such as problem solving, deviation of perception, feeling self-guilty and requesting help from others, with better compatibility and reduction of physical and psychological symptoms^5^. Castellano et al., in cancer adolescent survivors, found that using the problem-solving method, which aimed at detecting the source of stress, was accompanied by better physical and mental health, while social adaptation and avoidance coping led to the reduction of mental health^7^. The results of various studies indicate that children with chronic diseases cope maladaptive with the disease and consequent conditions and their quality of life is affected ^8^^-^^9^^-^^10^. On the other hand, there are few studies on the relationship between quality of life and coping styles in adolescents with thalassemia. Therefore, this study aimed to determine the relation between different aspects of quality of life and coping styles in adolescents with thalassemia in comparison with a healthy group. 

## MATERIALS AND METHODS

 The present study is a case-control, two-group and single-step survey which was conducted in an educating Hospital in Isfahan. Most of the adolescents with thalassemia were referred to this hospital, so that the possibility of the samples' follow-up increased. In the healthy group, the sampling was done among the healthy adolescents who lived in the same neighborhood as thalassemic adolescents lived. For ethical considerations, ethical approval was obtained from the Research and Technology Deputy of the Isfahan University of Medical Sciences (No: IR.MUI.REC.1395.2.074). Written informed consent was also obtained from participants to conduct this study. The criteria for the thalassemic adolescents to enter this research included the diagnosis of thalassemia with the approval of a specialist physician, age range of 11 to 21 years (due to more access to samples), lack of a diagnosed mental illness (referring to and checking out the patient's records), ability to answer questions and not having experienced a stressful incident like the death of a friend or family member^11^. All healthy adolescents who were matched by age, sex and geographical location with thalassemic adolescent*s *as well as all other criteria mentioned above for thalassemic adolescents, except having thalassemia (according to the patient's claim) were included in the healthy group. 

The exclusion criteria included lack of consent for participation in the study and completion of the questionnaires.

In this study, sample size was calculated as 100 in each group based on


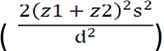


, where Z1 is the confidence coefficient of 0.95% i.e. 1.96, Z2 is the statistical power of 0.80% i.e. 0.84, S is an estimation of the standard deviation of the score of each one of the dimensions of quality of life and copying styles in two groups, D is the least difference of mean score in each one of the dimensions of quality of life and copying styles in two groups S (0.4), the number of samples was considered to be 100. The method used to implement this study was that the researcher, after obtaining a written permission from the research deputy of the Isfahan University of Medical Sciences, attended the research environment and after explaining the research goals to the supervisor of the department, was allowed to attend the ward and complete the questionnaires through simple sampling on days when adolescents were supposed to receive blood.

In the healthy group, 100 adolescents who lived in the same neighborhood as thalassemic adolescents (in order to match the variables of age, gender, and socioeconomic status) and had the appropriate conditions to enter the study were randomly selected. The researcher who was provided with the exact address of thalassemic adolescents attended the neighborhood and randomly selected the samples and completed the questionnaire.

Data was collected by Endler and Parker's coping style questionnaire and a WHOQOL-BREF questionnaire. The coping styles questionnaire included 48 questions, with Likert's five options, ranking from never^1^ to very much^5^. This questionnaire was used to examine three methods of stress coping which include task-oriented, emotion-oriented and avoidance-oriented coping styles, and each one of the behaviors which obtains a higher score in the scale is considered as the preferred coping style of the individual. This questionnaire includes 3 main dimensions of task-oriented (16 questions), emotion-oriented (16 questions) and avoidance-oriented (16 questions) coping behaviors ^12^. To measure the quality of life, the World Health Organization's quality of life questionnaire, which includes 26 questions in Likert scale of five options, was used. One question is about the general feeling of the person and the rest of the questions examine the person's feelings and behavior in the last two weeks in the physical, psychological, social and environmental dimensions. In this questionnaire each question has a score of 0 to 4, the score 0 indicates the worst and the 4 indicates the best quality of life status ^11^^, ^^13^. The psychometric properties of the Iranian version of the World Health Organization's quality of life questionnaire have shown that this tool can be used in Iran as well, so that the intra-cluster correlation index of the questionnaire was re-tested in two weeks in four domains from 0.75% to 0.84% ^11^^, ^^14^. In the Endler and Parker's coping style questionnaire, Cronbach's alpha coefficients were obtained for task, avoidance and emotion-oriented styles (78%, 73%, and 88%) respectively ^12^. The use of this questionnaire in childhood and adolescence in Iran indicates the reliability of this questionnaire in these age ^15^^,^^16^. Data were analyzed by SPSS 20 using independent *t*-test, linear regression and correlation coefficients.

## Results

 According to the results of this study, 48% of the respondents were girls and 52% boys. The average age of the case group was 17.5 ± 2.98 and the control group was 16.04 ± 3.7. Most of the adolescents participating in the study (41%) had diploma and their fathers had passed the secondary school (29.5%), and their mothers had passed primary and high school (30%). Most of the fathers were self-employed (39.5%) and the mothers were housewives (86.5%). Also, most of the adolescents were the first child of a 4 or 5-member-families (45%).

Independent t-test and one way ANOVA showed that there was a significant difference between the mean total score of quality of life and the psychological and environmental dimensions of quality of life in two groups of thalassemic adolescents and the healthy ones with (P <0.05), meaning that thalassemic adolescents have a lower quality of life, mental health and environmental relationships than healthy ones (Table 1). Also, independent t-test and one-way ANOVA showed that the mean scores of emotion and avoidance-oriented coping styles in thalassemic adolescents are lower than healthy ones. Thalassemic adolescents, compared with healthy ones, use emotion and avoidance-oriented coping styles in a more negative and inappropriate way (P<0.01) (Table 2).

Pearson correlation test showed that there is a positive and significant relationship between the mean score of social dimension of quality of life and emotion-oriented coping style (p <0.05), this means that increase use the emotion-oriented coping style, social relationships in adolescents' with thalassemic increase. However, there is a negative and significant relationship between the mean score of total quality of life and physical dimension and emotion-oriented coping style with (p = 0.05) in thalassemic adolescents. On the other hand, there is a direct and significant relation between the mean score of total quality of life and social dimension and task-oriented coping style in thalassemic adolescents with (p = 0.05) and physical health with (p = 0.01) (Table 3).

**Table 1:** The comparison of different aspects of quality of life between two groups

**Variable**	**Group **	**Mean ± SD**	**T **	**F**	**MS**	**DF**	**P**
Quality of life	Control Case	57.55±11.40 53.11±13.33	2.53^*^	6.41^*^	986.76	1	0.012
Physical healthy	Control Case	64.51±15.23 61.19±17.28	1.64^ n.s^	2.07^ n.s^		1	0.15
Mental healthy	Control Case	47.64±13.81 40.27±15.15	3.59^**^	12.9^**^	551.12	1	0.00
Social relationship	Control Case	62.20±20.36 60.06±21.06	0.73^ n.s^	0.53^ n.s^		1	0.46
Environmental relationship	Control	55.86±15.48	2.22^*^	4.95^*^	2715.84	1	0.02

(N=200, df=198) (** Significance at 1% level, * Significance at 5% level and MS insignificant)

**Table 2:** The comparison of different aspects of coping style between two groups

**variable**	**group**	**Mean ± SD**	**T **	**F**	**MS**	**DF**	**P**
Problem solving	control	53.85±9.36	-0.48	0.231	18.00	1	0.63
	Case	54.45±8.26					
Emotional	control	47.74±9.80	2.68^**^	7.19^**^	722.00	1	0.008
	Case	43.94±10.22					
Avoidance	control	51.94±8.01	2.65^**^	7.05^**^	420.50	1	0.009
	Case	49.04±7.40					

(N=200, df=198) (** Significance at 1% level, * Significance at 5% level and MS insignificant)

**Table 3:** Relationship between different aspects of quality of life with coping style

**Variable**	**group**		**Physical**	**Mental**	**Social**	**Environmental**	**Quality of life**
Emotional	Control	r	0.02	0.12	-0.16	-0.12	-0.08
		p	0.12	0.20	0.10	0.22	0.40
	Case	r	-0.25^**^	0.24	0.36^**^	-0.21	-0.35^**^
		p	0.001	0.23	0.000	0.13	0.000
Problem-solving	Control	r	0.22	-0.04	0.04	0.15	0.13
		p	0.12	0.63	0.63	0.12	0.18
	Case	r	0.26^**^	0.15	0.20^*^	0.15	0.25^*^
		p	0.001	0.12	0.04	0.12	0.011
Avoidance	Control	r	0.05	-0.002	0.20	0.01	-0.06
		p	0.59	0.98	0.33	0.89	0.50
	Case	r	0.13	0.17	0.25	0.11	0.27
		p	0.16	0.09	0.11	0.25	0.22

r= Pearson correlation coefficient, p= significance level, * significance at 1% level (p = 0.01), ** significance at 5% level (p = 0.05)

## Discussion

 The purpose of this study was to determine the relationship between different aspects of quality of life and coping styles in adolescents with thalassemia and healthy group. The results of this study showed that the quality of life of in adolescents with thalassemia in the psychological and environmental dimensions was lower than the healthy group. In this regard, the results of Hakeem et al. showed that the mean score of the mental health of adolescents with B-thalassemia was lower than that of healthy subjects, so that adolescents with thalassemia suffered from higher levels of depression and anxiety, which caused a significant reduction in their mental health^17^. The results of González-Echevarría et al. (2018), showed that adolescents with endometriosis higher levels of depression and anxiety than the control group, which directly affected their quality of life ^18^. The study by Gharaibeh et al. in Jordanian children with thalassemia found that, although the patients' quality of life was reduced in all dimensions, the lowest amount was related to school performance and peer communication^18^. In general, it can be said that factors such as fatigue following chronic anemia, the need for continuous blood transfusion and admission to treatment centers, impaired school performance due to frequent absences and depression affect these adolescents' quality of life ^19^^, ^^17^.

Other results of the present study indicate that adolescents with thalassemia were more likely to use emotion and avoidance-oriented coping styles than control group. In this regard, the results of the Smorti's study on cancer survivors indicated that adolescents were more likely to use avoidance-oriented coping style^20^. Li et al. Also found out that Chinese children with cancer were more likely to use emotion-oriented strategies than task-oriented ones^21^. The results of the study by Dellenmark-Blom et al. (2016), suggested that avoidance-oriented coping style, which includes having thoughts and actions to avoid situations and problems and hiding the emotions created by the disease, is the second common method for adolescents with Esophageal atresia in dealing with stressful situations ^22^. The results of the study by Yi-Frazier et al. showed that for the boys with diabetes type 1, the avoidance-oriented coping style was used as an emotion-oriented style and it was accompanied by fewer problems and better interaction with the medical personnel and other people related to that disease. The difference between the results of the present study and the results of the study by Yi-Frazier et al. could be the type of disease studied. While the present study was conducted on adolescents with chronic thalassemia, Yi-Frazier et al. was conducted on adolescents with acute diabetes. In this way, avoidance strategy is a relieving style which reduces the stress and anxiety and subsequently can reduce some mental health problems ^23^. 

The results of this study on the relationship between each dimension of quality of life and coping styles showed that when the emotion-oriented coping style was used more, the total quality of life and physical health of patients decreased, and when the emotion-oriented coping style was increasingly used, the social relationships of adolescents with thalassemia increased. In this regard, the results of a research by Sposito et al. showed that children with cancer who developed their spiritual beliefs showed more effective adaptation strategies and more flexibility to adapt to complications ^24^. Also, the results of Batista et al. (2015) in relation to coping styles in children with epilepsy showed that children with epilepsy used the emotion-oriented adaptation more commonly due to recognizing the disease as an uncontrollable situation^25^. However, contrary to the results of this study about the increasing use of emotion-oriented coping style and increasing social relationships among adolescents, Raheel's study showed that a large number of girls were more likely to use emotion-oriented coping style in stressful situations which led to their isolation, depression and growing stress ^26^. The difference between the results of this study and the mentioned one is due to the type of emotion-oriented coping style which has been used. In the present study, it seems that children, faced with stressors, tried to eliminate the stressor through expressing their feeling, which led them to improve their relationship with those around them.

Concerning the use of task-oriented coping style and the quality of life in adolescents with thalassemia, the results indicated a significant relationship between the use of this coping style and the total quality of life, social relationships and physical health of adolescents with thalassemia. The results of Castellano et al. (2013) in cancer survivors showed that adaptation methods, such as problem solving, the search for relieving ways, and trying to focus on positive issues, were associated with a better quality of life^7^. The results of Barberis et al. (2017) in dialysis patients showed that task-oriented adaptation strategies, in contrast to emotion-oriented adaptation strategies, like avoidance, positively impacted the quality of life of dialysis patients ^27^. To explain the results, it can be said that problem solving skills, with emphasis on cognitive and behavioral aspects, act as a shield against negative events. In other words, the patient instead of making avoidance decisions, decide decisively and after reviewing his past, identifies his weaknesses and strengths when faced with obstacles, he also examines the other ways ^28^. On the other hand, because in this coping style the patient after diagnosing the problem, evaluating the situation and considering the goals, is after the best options the implementation of the decision made can be done in the best way by him^29^. 

**Study limitation**

The most important limitation of the present study was to complete the questionnaire in the healthy group. In some cases, parents were not allowed to complete the questionnaires; therefore, the time to complete the questionnaires in the healthy group was prolonged.

## CONCLUSION

 According to the results of this study, there is a significant relationship between some dimensions of quality of life and coping styles in adolescents with thalassemia. Thus, the need for recognizing effective adaptation strategies and their utility in relation to health outcomes in adolescents with thalassemia is revealed by the health care personnel. Additionally, teaching adolescents and their families how to cope with stressors and preparing them to enter their youth can be of great importance. 
